# Young Men’s Christian Association

**Published:** 1875-11

**Authors:** 


### THE YOUNG MEN'S CHRISTIAN ASSOCIATION.

This institution is doing a vast deal of good in this city, and we are glad to learn that new members are flocking in. The cost is but five dollars per year, which gives the member the privileges of the reading-room, library, literary society, gymnasium, bowling-alley, baths, evening classes in German, French, Spanish, Book-Keeping, Writing, and Vocal Music; and admission for himself, with the privilege of bringing a lady, to the Receptions (second Monday of each month), and to the Members' Lectures. All young men of good moral character are eligible to membership.

We have attended several of the lectures given before the Association this fall, and have been highly edified thereby. The best talent is employed, and the large hall is always filled with intelligent listeners. Every young man should secure the advantages offered by the Association.

